# Impact of duration of maintenance immunotherapy on the prognosis of locally advanced non-small cell lung cancer treated with chemoradiotherapy

**DOI:** 10.3389/fonc.2025.1580396

**Published:** 2025-07-21

**Authors:** HuiQi Fan, Song Guan, Kai Ren, Xue Li, Jun Wang, Nan Bi, Lujun Zhao

**Affiliations:** ^1^ Department of Radiation Oncology, Tianjin Medical University Cancer Institute & Hospital, National Clinical Research Center for Cancer, Tianjin’s Clinical Research Center for Cancer, Key Laboratory of Cancer Immunology and Biotherapy, Tianjin, China; ^2^ Department of Radiation Oncology, Beijing Tuberculosis and Thoracic Tumor Research Institute/Beijing Chest Hospital, Cancer Research Center, Capital Medical University, Beijing, China; ^3^ Department of Radiotherapy, The Fourth Hospital of Hebei Medical University, Hebei Clinical Research Center for Radiation Oncology, Shijiazhuang, China; ^4^ Department of Radiation Oncology, National Cancer Center/National Clinical Research Center for Cancer/Cancer Hospital, Chinese Academy of Medical Sciences and Peking Union Medical College, Beijing, China

**Keywords:** non-small cell lung cancer, consolidation immunotherapy, chemoradiotherapy, immunotherapy duration, disease prognosis

## Abstract

**Purpose:**

Chemoradiotherapy combined with consolidation immunotherapy is the standard of care for unresectable stage III non-small cell lung cancer; however, the optimal number of cycles of consolidation immunotherapy remains unknown. This study aimed to investigate the optimal duration of consolidation immunotherapy after chemoradiotherapy.

**Materials and methods:**

We conducted a real-world, multicenter, retrospective study of patients with unresectable stage III non-small cell lung cancer who underwent consolidation immunotherapy between February 2018 and December 2022 following chemoradiotherapy. The inclusion criteria were as follows: (1) age ≥18 years and Karnofsky Performance Scale (KPS) score ≥70; (2) histopathologically confirmed stage III non-small cell lung cancer; and (3) received consolidation immunotherapy after chemoradiotherapy. The exclusion criteria were as follows: (1) patients with EGFR or ALK gene mutations; (2) history of other cancers; (3) tumor progression prior to immunotherapy; (4) immunotherapy concurrently with chemoradiotherapy; and (5) discontinuation of immunotherapy due to detection of disease progression. Univariate analysis was performed via the Cox proportional risk model. The correlations between immunotherapy duration and survival outcomes were determined via Kaplan–Meier and log-rank analyses. The study endpoints in this study were overall survival (OS) and progression-free survival (PFS).

**Results:**

The median number of cycles of consolidation immunotherapy was 10 (interquartile range: 4, 19). The 1-year OS rates were 91.3% and 100% for patients with ≤ 10 and >10 cycles of immunotherapy, respectively (P<0.001), and the 1-year PFS rates were 53.4% and 98.4%, respectively (P<0.001). And the 1-year OS rates of patients with ≤ 4, > 4 - ≤ 10, > 10 - ≤ 19, and >19 cycles of consolidation immunotherapy were 89.1%, 93.8%, 100%, and 100%, respectively (≤ 4 vs. 4-10: p=0.068; 4–10 vs. 10-19: p=0.023; 10–19 vs. >19: p= 0.435). The 1-year PFS rates were 48.3%, 59.4%, 96.7%, and 100%, respectively (≤ 4 vs. 4-10: P=0.394; 4–10 vs. 10-19: P=0.002; 10–19 vs. >19: P=0.019). In radiotherapy modality subgroup analyses (stratified by histology, immunotherapy type, and concurrent chemoradiotherapy), immunotherapy cycle number significantly predicted prognosis in all subgroups (all p < 0.05).

**Conclusion:**

In patients with locally advanced non-small cell lung cancer who received consolidation immunotherapy after chemoradiotherapy, the number of cycles of immunotherapy was significantly associated with prognosis. These results need to be further validated in a large prospective study.

## 1 Introduction

Lung cancer is the leading cause of cancer-related deaths worldwide, with non-small cell lung cancer accounting for approximately 80% of all lung cancer cases and more than one-third of non-small cell lung cancer cases being in locally advanced stages (stage III) (1–3). Currently, the proportion of advanced non-small cell lung cancer cases is increasing annually (4, 5). Concurrent chemoradiotherapy is the treatment of choice for unresectable non-small cell lung cancer (2). The emergence of immunotherapy has changed the treatment options for patients with stage III non-small cell lung cancer. In the PACIFIC trial, the use of durvalumab after chemoradiotherapy produced significant and long-term benefits in terms of overall survival (OS) and progression-free survival (PFS) compared with placebo. The median OS was 47.5 months (vs. 29.1 months), and the 5-year OS rate was 42.9% (vs. 33.4%) (6–9). For patients with unresectable stage III non-small cell lung cancer, simultaneous chemoradiotherapy plus consolidation immunotherapy has become the standard of care (6, 10–12).

However, the optimal number of cycles of consolidated immunotherapy has not been determined. Prolonged immunotherapy carries a large financial burden; therefore, studies on the optimal duration of immunotherapy constitute one of the current research hotspots (12–16). In the Checkmate-153 trial, patients who stopped immunotherapy at 1 year had significantly shorter PFS and OS than patients who continued immunotherapy (17). However, a prolonged duration of immunotherapy may increase the cost of treatment. Therefore, it is important to identify the optimal duration of consolidation immunotherapy is important. Herein, we conducted a retrospective study to explore the relationship between immunotherapy duration and disease prognosis.

## 2 Materials and methods

### 2.1 Patient selection

This study conformed to the provisions of the Declaration of Helsinki (as revised in 2013) and was approved by the institutional medical ethics committee (No. bc2022212).

This multicenter retrospective study included patients with unresectable stage III non-small cell lung cancer who received consolidation immunotherapy after chemoradiotherapy at Tianjin Cancer Hospital, the Chinese Academy of Medical Science Cancer Institute & Hospital, Beijing Chest Hospital, and the Fourth Hospital of Hebei Medical University between February 2018 and December 2022. The inclusion criteria were as follows: (1) age ≥18 years and Karnofsky Performance Scale (KPS) score ≥70; (2) histopathologically confirmed stage III non-small cell lung cancer; and (3) received consolidation immunotherapy after chemoradiotherapy. The exclusion criteria were as follows: (1) patients with epidermal growth factor receptor (EGFR) mutations or an anaplastic lymphoma kinase (ALK) rearrangement; (2) a history of other cancers; (3) tumor progression prior to immunotherapy; (4) concomitant immunotherapy with chemoradiotherapy; and (5) discontinuation of immunotherapy due to detection of disease progression. Baseline characteristics and treatment information were collected to determine the histologic type and stage of the cancers according to World Health Organization (WHO) criteria (18) and the eighth edition of the lung cancer classification (19). The following variables were collected from the patients: age, sex, pathology, stage, radiotherapy dose, smoking status, Eastern Cooperative Oncology Group Performance Status (ECOG-PS), and type of chemoradiotherapy.

In this study, case data were collected from 334 patients with locally advanced non-small cell lung cancer; 138 of these patients were treated with a consolidation immunotherapy regimen only. Additionally, only patients who discontinued consolidation immunotherapy before disease progression or continued immunotherapy after progression were included in this study; five patients discontinued immunotherapy due to disease progression and were thus not included. Ultimately, a total of 133 patients were included in the analysis.

### 2.2 Treatment regimen

Each patient's chemoradiation therapy regimen was determined by a team of radiation oncologists, who use different chemotherapy regimens for patients on the basis of their tissue type and individual clinical status. Common chemotherapy regimens include paclitaxel with cisplatin or carboplatin, pemetrexed with cisplatin or carboplatin, etoposide with cisplatin or carboplatin, docetaxel with cisplatin, and albumin-bound paclitaxel with cisplatin. All patients were treated with PD-1/L1 antibodies, such as atezolizumab (N=2), sugemalimab (N=23), camrelizumab (N=9), durvalumab (N=56), nivolumab (N=2), pembrolizumab (N=3), sintilimab (N=17), tislelizumab (N=10) or toripalimab (N=11), based on the discussion results between the treatment physicians and the patients after the completion of chemoradiotherapy.

### 2.3 Study endpoint

The endpoints of this study included PFS and OS. PFS was defined as the time from the diagnosis of lung cancer to disease progression, last follow-up or death. OS was defined as the time from diagnosis to death or last follow-up. Patients were followed every 3 months for 2 years and every 6 months thereafter, including clinical evaluation, CT or PET-CT, and related clinical examinations.

### 2.4 Statistical analysis

Patient characteristics and correlations with survival outcomes were examined via Cox proportional risk models. The duration of immunotherapy was divided into quartiles, and Kaplan–Meier and log-rank analyses were used to determine the correlation between immunotherapy duration and survival outcomes. A P value < 0.05 was considered to indicated statistical significance. Statistical analyses were performed via IBM SPSS Statistics 27.

## 3 Results

### 3.1 Baseline characteristics

A total of 133 patients with unresectable stage III non-small cell lung cancer were included in this study. The baseline characteristics and their correlations with OS and PFS in the univariate analysis are shown in **Table 1**. As shown in **Table 1**, the number of immunotherapy cycles was significantly correlated with OS (P < 0.001) and PFS (P < 0.001).

**Table 1:** Patient characteristics.

Characteristics	N=133	OS		PFS	
		P	HR (95%CI)	P	HR (95%CI)
Sex		0.113	0.200 (0.027-1.467)	0.550	0.799 (0.382-1.669)
Male*	116				
Female	17				
Age**		0.763	1.007 (0.963-1.053)	0.915	0.998 (0.969-1.029)
Range	32-79				
Median	63				
WHO Histology		0.446	0.746 (0.351-1.585)	0.937	1.020 (0.629-1.654)
Squamous*	81				
Non-Squamous	52				
Stage		0.242		0.821	
IIIA*	47				
IIIB	61	0.861	1.079 (0.461-2.526)	0.865	1.048 (0.613-1.791)
IIIC	25	0.133	2.034 (0.805-5.136)	0.540	1.231 (0.633-2.394)
Radiation Dose**		0.064	0.897 (0.799-1.006)	0.039	0.960 (0.924-0.998)
Range	45-66				
Median	60.2				
Smoking		0.354	1.643 (0.575-4.698)	0.831	1.068 (0.584-1.953)
Never*	26				
Former/Current	107				
ECOG		0.326		0.830	
0*	16				
1	115	0.135	4.580 (0.624-33.599)	0.542	1.276 (0.582-2.796)
2	2	0.985	/	0.971	/
CRT modality		0.542	1.273 (0.586-2.768)	0.833	0.948 (0.577-1.557)
sCRT*	43				
cCRT	90				
Consolidation Treatment Timing***		0.422	0.968 (0.893-1.048)	0.796	0.995 (0.960-1.032)
Range	0-61.3				
Median	5.0				
Immunotherapy drugs		0.480	1.305 (0.624-2.730)	0.942	1.018 (0.629-1.646)
PD-1 inhibitor*	52				
PD-L1 inhibitor	81				
Immune Cycle**		<0.001	0.857 (0.795-0.924)	<0.001	0.913 (0.884-0.943)
Range	1-63				
Median	10				

*Control group in cox regression analysis.

**Cox regression analysis for continuous variables.

***Time(weeks) difference from the end of radiotherapy to the start of consolidation immunotherapy.

### 3.2 Impact of duration of consolidated immunotherapy on prognosis

**Figure 1:**
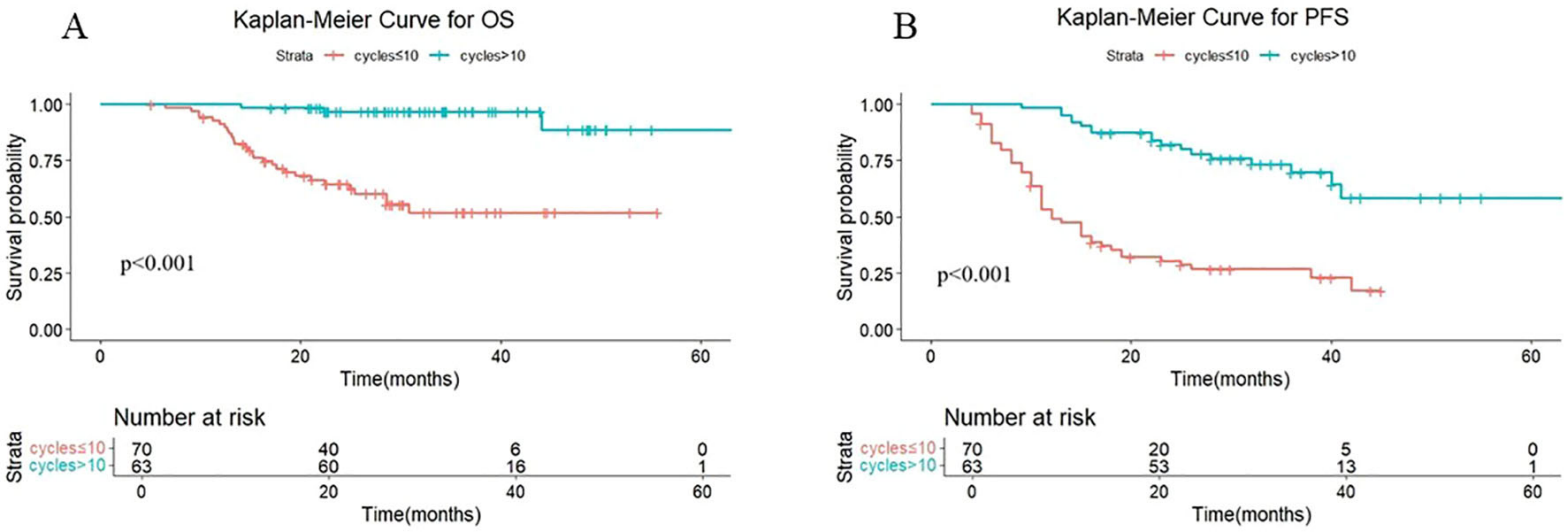
**(A)** Kaplan-Meier Curve for OS in two groups. **(B)** Kaplan-Meier Curve for PFS in two groups.

We divided patients into two groups based on the median number of cycles of immunotherapy: patients with >10 cycles or patients with ≤10 cycles. As shown in **Figure 1**, the median OS was not reached either in patients with >10 cycles or in those with ≤10 cycles, with median PFS times of not reached and 12.4 months, respectively. The 1-year OS rates were 100% and 91.3% (P < 0.001), and the 1-year PFS rates were 98.4% and 53.4%, respectively (P < 0.001).

**Figure 2:**
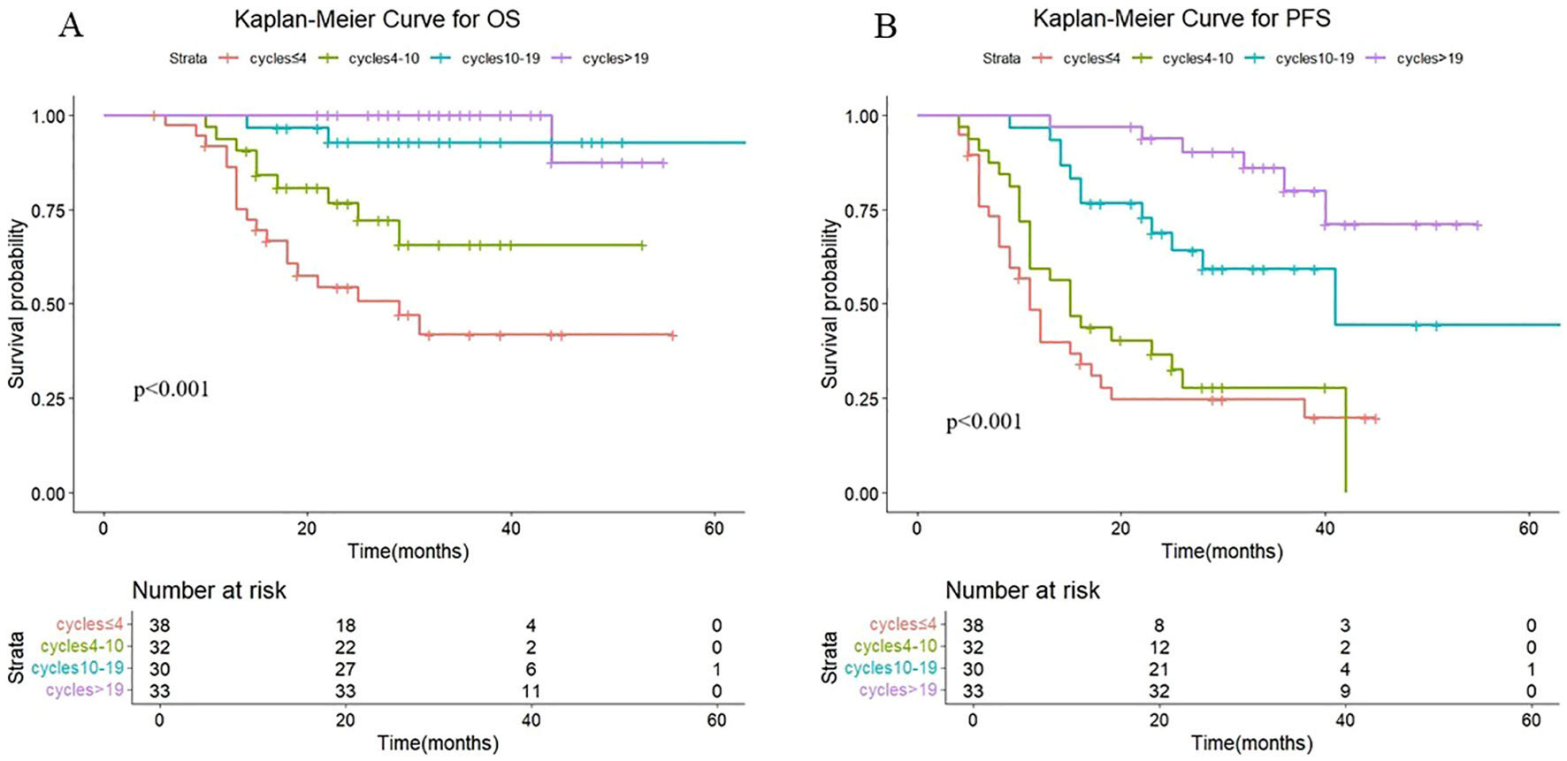
**(A)** Kaplan-Meier Curve for OS in four groups. **(B)** Kaplan-Meier Curve for PFS in four groups.

The quartiles for the number of consolidated immunotherapy cycles are shown in **Figure 2** and **Table 2**. There were significant differences in both OS and PFS between the quartiles (P<0.001). When the effect of the number of immunotherapy cycles on prognosis was compared between two adjacent groups, OS increased with an increasing number of consolidated immunotherapies (≤4 vs. 4-10: P=0.068; 4–10 cycles vs. 10–19 cycles: p=0.023; 10–19 vs. >19: P=0.435). PFS also increased with an increasing number of consolidated immunotherapies (≤4 vs. 4-10: P=0.394; 4–10 vs. 10-19: P=0.002; 10–19 vs. >19: P=0.019).

**Table 2:** OS and PFS in patients with different numbers of cycles.

Cycles	Number	Median OS (month)	1-year OS (%)	2-year OS (%)	P	Median PFS (month)	1-year PFS (%)	2-year PFS (%)	P
≤ 4	38	28.6	89.1	54.2	<0.001^#^	11.2	48.3	24.8	<0.001^#^
4-10	32	NR	93.8	76.4	0.068^*^	14.9	59.4	36.3	0.394*
10-19	30	NR	100	92.6	0.023**	41.1	96.7	68.5	0.002**
>19	33	NR	100	100	0.435***	NR	100	93.5	0.019***

^#^Comparison of the 4 groups. *4–10 cycles vs. ≤4 cycles. **10–19 cycles vs.-10 cycles. *** >19 cycles vs.-19 cycles.

### 3.3 Subgroup analysis of the number of immune cycles on patient prognosis (cCRT vs sCRT)

**Figure 3:**
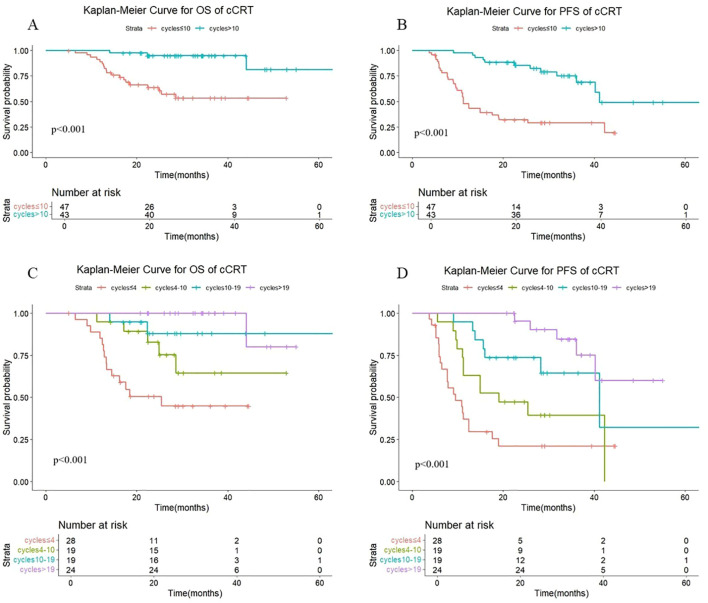
**(A)** Kaplan-Meier Curve for OS of cCRT in two groups. **(B)** Kaplan-Meier Curve for PFS of cCRT in two groups. **(C)** Kaplan-Meier Curve for OS of cCRT in four groups. **(D)** Kaplan-Meier Curve for PFS of cCRT in four groups.

Additional subgroup analyses by radiotherapy modality were conducted to assess the association between the number of consolidation immunotherapy cycles and patient prognosis. As shown in **Figure 3**, a statistically significant difference in both OS (P < 0.001) and PFS (P < 0.001) was observed between patients receiving >10 versus ≤10 consolidation immunotherapy cycles among those treated with concurrent radiotherapy. The median OS was not reached either in patients with >10 cycles or in those with ≤10 cycles, with median PFS times of 41.1 months and 11.2 months, respectively. The 1-year OS rates were 100% and 89.1%, and the 1-year PFS rates were 97.7% and 47.9%, respectively. The quartiles for the number of consolidated immunotherapy cycles are shown in **Figure 3** and **Table 3**. There were significant differences in both OS and PFS between the quartiles (P<0.001). When the effect of the number of immunotherapy cycles on prognosis was compared between two adjacent groups, OS increased with an increasing number of consolidated immunotherapies (≤4 vs. 4-10: P=0.056; 4–10 cycles vs. 10–19 cycles: p=0.255; 10–19 vs. >19: P=0. 0.280). PFS also increased with an increasing number of consolidated immunotherapies (≤4 vs. 4-10: P=0.074; 4–10 vs. 10-19: P=0.081; 10–19 vs. >19: P=0.075).

**Table 3:** OS and PFS in cCRT-patients and sCRT-patients with different numbers of cycles.

CRT modality	Cycles	Number	Median OS (month)	1-year OS (%)	2-year OS (%)	P	Median PFS (month)	1-year PFS (%)	2-year PFS (%)	P
cCRT	≤4	28	25.4	85.2	50.6	<0.001#	9.5	37.1	21.2	<0.001#
	4-10	19	NR	94.7	82.8	0.056*	19.0	63.2	47.4	0.074*
	10-19	19	NR	100	88.0	0.255**	41.1	94.7	73.7	0.081**
	>19	24	NR	100	100	0.280***	NR	100	95.2	0.075***
sCRT	≤4	28	NR	100	66.7	<0.001#	15.6	80.0	34.3	<0.001#
	4-10	19	NR	92.3	67.7	0.774*	13.3	53.8	20.5	0.313*
	10-19	19	NR	100	100	0.044*	NR	100	72.7	0.008**
	>19	24	NR	100	100	/***	NR	100	88.9	0.142***

^#^Comparison of the 4 groups. *4–10 cycles vs. ≤4 cycles. **10–19 cycles vs.-10 cycles. *** >19 cycles vs.-19 cycles.

**Figure 4:**
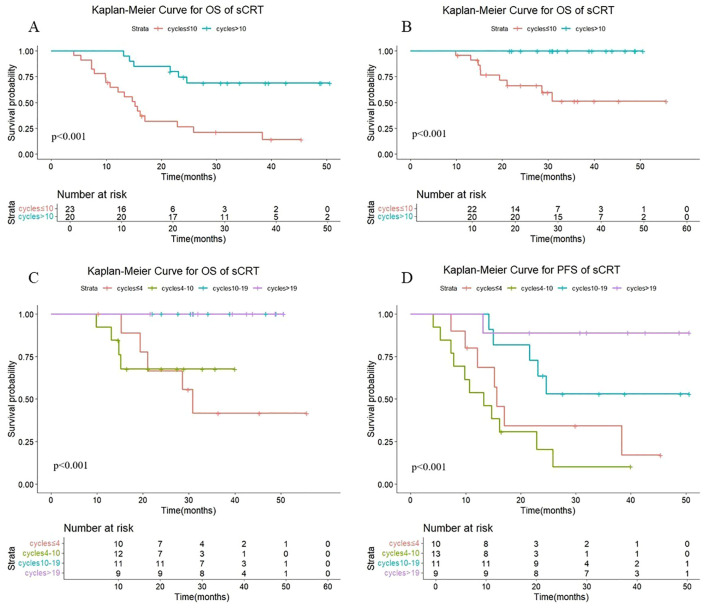
**(A)** Kaplan-Meier Curve for OS of sCRT in two groups. **(B)** Kaplan-Meier Curve for PFS of sCRT in two groups. **(C)** Kaplan-Meier Curve for OS of sCRT in four groups. **(D)** Kaplan-Meier Curve for PFS of sCRT in four groups.

As shown in **Figure 4**, there were statistically significant differences in OS (P < 0.001) and PFS (P < 0.001) in sequential radiotherapy patients with different numbers of cycles of consolidation immunization (>10 vs. ≤10). The median OS was not reached either in patients with >10 cycles or in those with ≤10 cycles, with median PFS times of not reached and 15.2 months, respectively. The 1-year OS rates were 100% and 95.7%, and the 1-year PFS rates were 100% and 64.9%, respectively. The quartiles for the number of consolidated immunotherapy cycles are shown in **Figure 4** and **Table 3**. There were significant differences in both OS and PFS between the quartiles (P<0.001). When the effect of the number of immunotherapy cycles on prognosis was compared between two adjacent groups, patients receiving 4–10 cycles showed significantly different OS (P = 0.044) and PFS (P = 0.008) outcomes compared to those receiving 10–19 cycles. No other intergroup comparisons showed statistically significant prognostic differences.

### 3.4 Subgroup analysis of immunotherapy types

**Figure 5:**
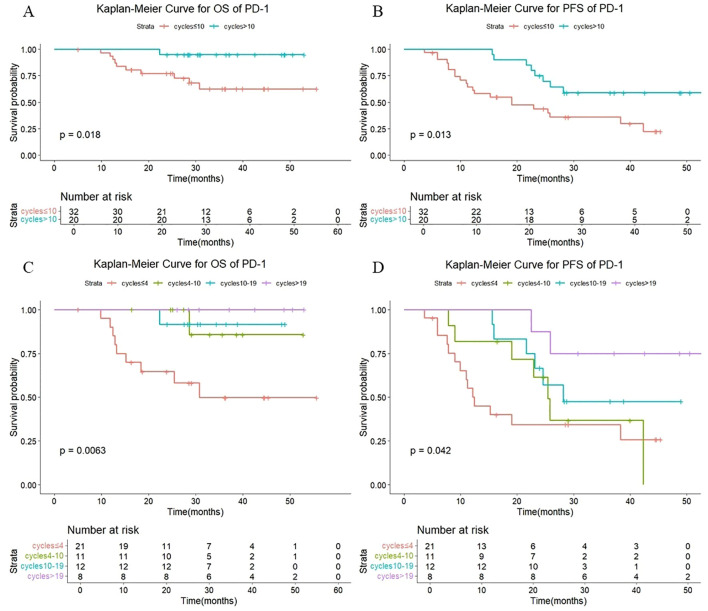
**(A)** Kaplan-Meier Curve for OS of PD-1 in two groups. **(B)** Kaplan-Meier Curve for PFS of PD-1 in two groups. **(C)** Kaplan-Meier Curve for OS of PD-1 in four groups. **(D)** Kaplan-Meier Curve for PFS of PD-1 in four groups.

As shown in **Figure 5**, among those treated with PD-1 antibody, a statistically significant difference in both OS (P =0.018) and PFS (P =0.013) was observed between patients receiving >10 versus ≤10 consolidation immunotherapy cycles. The median OS was not reached either in patients with >10 cycles or in those with ≤10 cycles, with median PFS times of not reached and 24.8 months, respectively. The 1-year OS rate was 100% vs. 93.5%, and the 1-year PFS rate was 100% vs. 64.6%, respectively. The quartiles for the number of consolidated immunotherapy cycles are shown in **Figure 5** and **Table 4**. There were significant differences in both OS and PFS between the quartiles (P<0.05). The patients receiving ≤4 cycles showed significantly worse OS (P = 0.016) outcomes compared to those receiving 4–10 cycles. No other intergroup comparisons showed statistically significant prognostic differences.

**Table 4:** OS and PFS in PD-1-patients and PD-L1-patients with different numbers of cycles.

Immunotherapy drugs	Cycles	Number	Median OS (month)	1-year OS (%)	2-year OS (%)	P	Median PFS (month)	1-year PFS (%)	2-year PFS (%)	P
PD-1 inhibitor	≤4	21	30.8	90	64.6	0.006^#^	12.4	55.1	34.4	0.042^#^
	4-10	11	NR	100	100	0.049*	25.4	81.8	61.4	0.422*
	10-19	12	NR	100	91.7	0.911**	28.2	100	66.7	0.450**
	>19	8	NR	100	100	0.414***	NR	100	87.5	0.223***
PD-L1 inhibitor	≤4	17	19.4	88.2	41.6	<0.001 #	9.5	40.3	13.4	<0.001 #
	4-10	21	NR	90.5	62.7	0.237*	11.1	47.6	23.8	0.349*
	10-19	18	NR	100	94.4	0.016**	41.1	94.4	72.2	0.002**
	>19	25	NR	100	100	0.827***	NR	100	96	0.151***

^#^Comparison of the 4 groups. *4–10 cycles vs. ≤4 cycles. **10–19 cycles vs.-10 cycles. ***>19 cycles vs.-19 cycles

**Figure 6:**
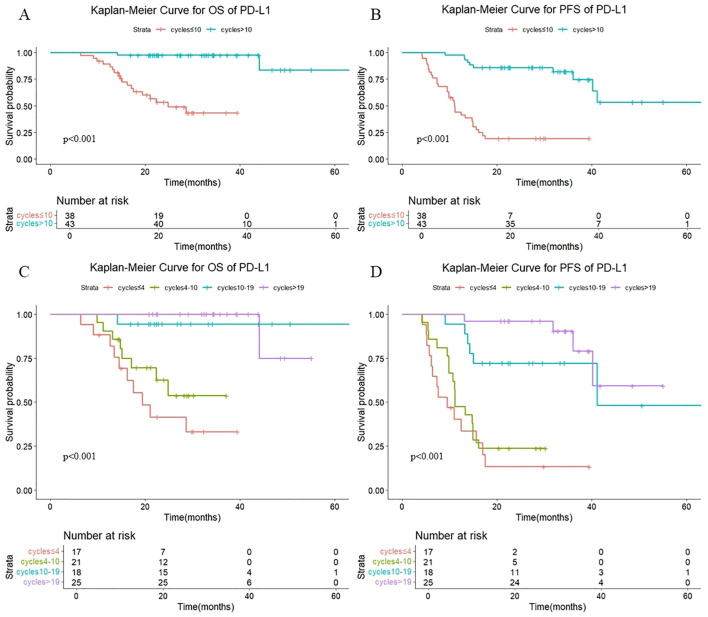
**(A)** Kaplan-Meier Curve for OS of PD-L1 in two groups. **(B)** Kaplan-Meier Curve for PFS of PD-L1 in two groups. **(C)** Kaplan-Meier Curve for OS of PD-L1 in four groups. **(D)** Kaplan-Meier Curve for PFS of PD-L1 in four groups.

As shown in **Figure 6**, in patients treated with PD-L1 antibody, there were also statistically significant differences in OS (P < 0.001) and PFS (P < 0.001) with different numbers of cycles of consolidation immunization (>10 vs. ≤10). The median OS was not reached either in patients with >10 cycles or in those with ≤10 cycles, with median PFS times of not reached and 11.0 months, respectively. The 1-year OS rate was 100% vs. 89.4%, and the 1-year PFS rate was 97.7% vs. 44.1%, respectively. The quartiles for the number of consolidated immunotherapy cycles are shown in **Figure 6** and **Table 4**. There were significant differences in both OS and PFS between the quartiles (P<0.001). The patients receiving 4–10 cycles showed significantly worse OS (P = 0.016) and PFS (P = 0.002) outcomes compared to those receiving 10–19 cycles. No other intergroup comparisons showed statistically significant prognostic differences.

### 3.5 Subgroup analysis of different pathology types

**Figure 7:**
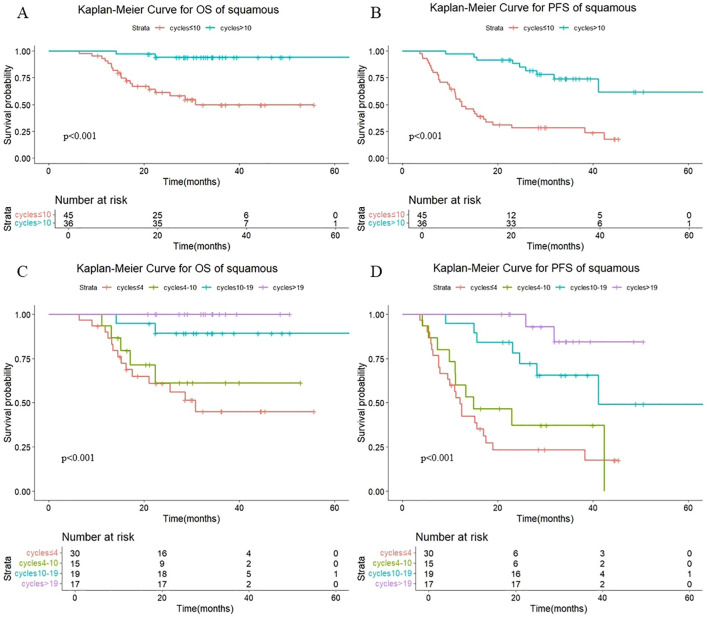
**(A)** Kaplan-Meier Curve for OS of squamous in two groups. **(B)** Kaplan-Meier Curve for PFS of squamous in two groups. **(C)** Kaplan-Meier Curve for OS of squamous in four groups. **(D)** Kaplan-Meier Curve for PFS of squamous in four groups.

As shown in **Figure 7**, a statistically significant difference in both OS (P <0.001) and PFS (P <0.001) was observed between patients receiving >10 versus ≤10 consolidation immunotherapy cycles among squamous patients. The median OS was not reached patients with >10 cycles and 30.8 mouths in those with ≤10 cycles, with median PFS times of not reached and 12.4 months, respectively. The 1-year OS rate was 100% vs. 91.0%, and the 1-year PFS rate was 97.2% vs. 55.2%, respectively. The quartiles for the number of consolidated immunotherapy cycles are shown in **Figure 7** and **Table 4**. There were significant differences in both OS and PFS between the quartiles (P<0.001). The patients receiving 4–10 cycles showed significantly worse PFS (P = 0.016) compared with those receiving 10–19 cycles. No other intergroup comparisons showed statistically significant prognostic differences.

**Figure 8:**
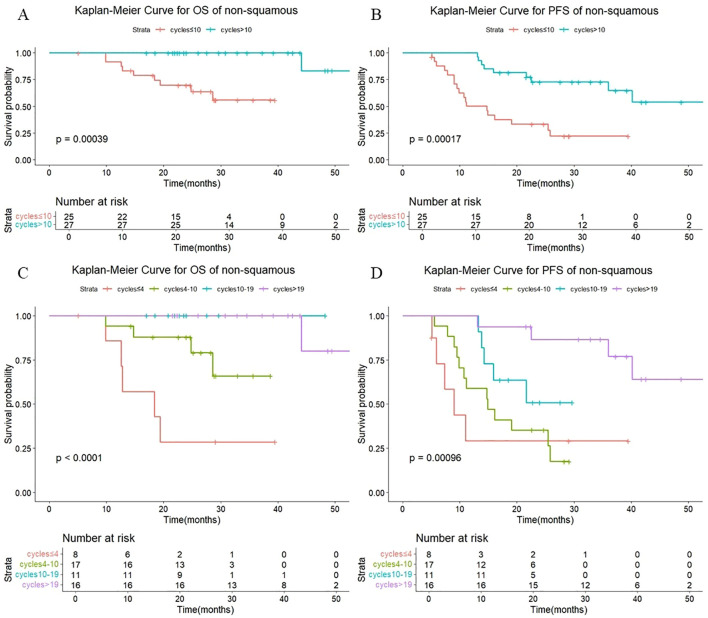
**(A)** Kaplan-Meier Curve for OS of non-squamous in two groups. **(B)** Kaplan-Meier Curve for PFS of non-squamous in two groups. **(C)** Kaplan-Meier Curve for OS of non-squamous in four groups. **(D)** Kaplan-Meier Curve for PFS of non-squamous in four groups.

As shown in **Figure 8**, there were also statistically significant differences in OS (P < 0.001) and PFS (P < 0.001) in non-squamous patients with different numbers of cycles of consolidation immunization (>10 vs. ≤10). The median OS was not reached either in patients with >10 cycles or in those with ≤10 cycles, with median PFS of not reached and 14.7 months, respectively. The 1-year OS rate was 100% vs. 91.7%, and the 1-year PFS rate was 100% vd. 50.1%, respectively. The quartiles for the number of consolidated immunotherapy cycles are shown in **Figure 8** and **Table 5**. There were significant differences in both OS and PFS between the quartiles (P<0.001). The patients receiving ≤4 cycles showed significantly worse OS (P = 0.024) compared with those receiving 4–10 cycles while receiving 10–19 cycles showed significantly worse PFS (P = 0.040) compared with those receiving >19 cycles. No other intergroup comparisons showed statistically significant prognostic differences.

**Table 5:** OS and PFS in squamous-patients and non-squamous-patients with different numbers of cycles.

WHO Histology	Cycles	Number	Median OS (month)	1-year OS (%)	2-year OS (%)	P	Median PFS (month)	1-year PFS (%)	2-year PFS (%)	P
Squamous	≤4	30	30.8	89.9	60.9	<0.001^#^	12.1	52.9	23.5	<0.001 #
	4-10	15	NR	93.3	61.3	0.490*	14.9	60.0	37.3	0.428*
	10-19	19	NR	100	89.2	0.059**	41.1	94.7	78.2	0.016**
	>19	17	NR	100	100	0.175***	NR	100	100	0.100***
Non-Squamous	≤4	8	18.4	85.7	28.6	<0.001^#^	9.0	29.2	29.2	<0.001 #
	4-10	17	NR	94.1	87.8	0.024*	14.9	58.8	35.3	0.629*
	10-19	11	NR	100	100	0.148**	NR	100	50.9	0.142**
	>19	16	NR	100	100	0.655***	NR	100	86.5	0.040***

^#^Comparison of the 4 groups. *4–10 cycles vs. ≤4 cycles. **10–19 cycles vs.-10 cycles. ***>19 cycles vs.-19 cycles.

## 4 Discussion

Currently, consolidation immunotherapy after chemoradiotherapy has become the standard of care for patients with unresectable stage III non-small cell lung cancer, but the optimal number of cycles of consolidation therapy is currently unclear. In this study, our treatment strategies are rigorously aligned with the most current clinical guidelines, including the 2023 European Society for Medical Oncology (ESMO) recommendations and the National Comprehensive Cancer Network (NCCN) Guidelines (Version 1.2, 2024). For complex cases, we employ a multidisciplinary team (MDT) approach to ensure optimal patient-centered decision-making. We investigated the role of the number of cycles of consolidation immunotherapy in unresectable stage III non-small cell lung cancer patients and reported that OS and PFS seem to increase with an increased number of consolidation immunotherapy cycles. However, the optimal number of immunotherapy cycles for patient treatment has yet to be determined.

Our retrospective analysis revealed that the survival benefit increased as patients underwent a greater number of cycles of immunotherapy. There was a significant increase in PFS among patients with >19 cycles of consolidation immunotherapy compared with those with 10–19 cycles; however, there was no significant increase in OS between these two subgroups. This findings was considered related to the short follow-up period and the small number of cases. Bryant et al. reported that, in patients with stage III non-small cell lung cancer, consolidation immunotherapy was less effective when the duration was less than 6 months; at a duration of 9 months, patients achieved a better survival benefit in terms of OS and PFS (20). These findings align with our study's comparison of >10 versus ≤10 immunotherapy cycles, where patients receiving >10 cycles demonstrated significantly better OS and PFS outcomes than those receiving ≤10 cycles. In addition, Shaverdian et al. analyzed the prognosis of 113 patients who received chemoradiotherapy plus consolidation immunotherapy and reported that patients who discontinued consolidation immunotherapy at an early stage had worse PFS than those who discontinued it at a later stage (21). This study, along with several others, has demonstrated that prolonged consolidation immunotherapy is associated with improved survival in patients.

Additionally, Li et al. found that prolonged immunotherapy consolidation improved 1-year PFS and OS rates in stage III NSCLC patients without increasing severe radiation pneumonitis incidence (16). While this study did not examine the relationship between consolidation immunotherapy cycle number and radiation injury, it did explore the association between cycle number and prognosis in sequential radiotherapy patients. The findings demonstrated that increased immunotherapy cycles improved outcomes in these patients, with those receiving 10–19 cycles showing significantly better prognosis than those receiving 4–10 cycles.

There are several limitations to this study. First, because it was a retrospective study, the treatment patterns were not fully standardized, and there was a large degree of heterogeneity. The relatively small number of patients affected the statistical validity of the results. The follow-up period was relatively short, and comparative judgments about overall survival time are still premature. Our findings suggest that patient outcomes may improve further as the number of cycles of consolidation immunotherapy increases. However, the optimal number of cycles of immune-consolidation therapy needs to be confirmed by further large-sample prospective studies.

## 5 Conclusion

In patients with locally advanced non-small cell lung cancer receiving consolidation immunotherapy after chemoradiotherapy, the number of cycles of immunotherapy was significantly associated with disease prognosis. These findings need to be further validated in a large prospective study.

## Data Availability

The raw data supporting the conclusions of this article will be made available by the authors, without undue reservation.
